# Acute Epigallocatechin-3-Gallate Supplementation Alters Postprandial Lipids after a Fast-Food Meal in Healthy Young Women: A Randomized, Double-Blind, Placebo-Controlled Crossover Study

**DOI:** 10.3390/nu12092533

**Published:** 2020-08-21

**Authors:** Alcides C. de Morais Junior, Raquel M. Schincaglia, Marisa Passarelli, Gustavo D. Pimentel, João F. Mota

**Affiliations:** 1 Clinical and Sports Nutrition Research Laboratory (LABINCE), Faculty of Nutrition, Federal University of Goiás (UFG), Goiania 74690-900, GO, Brazil; allsmorais@yahoo.com.br (A.C.d.M.J.); raquelms@outlook.com (R.M.S.); gdpimentel@gmail.com (G.D.P.); 2 Laboratório de Lípides (LIM 10), Hospital das Clínicas (HCFMUSP), Faculdade de Medicina da Universidade de Sao Paulo, Sao Paulo 05403-900, Brazil; m.passarelli@fm.usp.br; 3 Programa de Pós-Graduação em Medicina da Universidade Nove de Julho, Sao Paulo 01504-000, Brazil

**Keywords:** green tea, epigallocatechin, lipid profile, high-fat diet, fast food

## Abstract

A high-fat fast-food meal negatively impacts postprandial metabolism even in healthy young people. In experimental studies, epigallocatechin-3-gallate (EGCG), a bioactive compound present in green tea, has been described as a potent natural inhibitor of fatty acid synthase. Thus, we sought to evaluate the effects of acute EGCG supplementation on postprandial lipid profile, glucose, and insulin levels following a high-fat fast-food meal. Fourteen healthy young women 21 ± 1 years and body mass index 21.4 ± 0.41 kg/m^2^ were enrolled in a randomized, double-blind, placebo-controlled crossover study. Participants ingested capsules containing 800 mg EGCG or placebo immediately before a typical fast-food meal rich in saturated fatty acids. Blood samples were collected at baseline and then at 90 and 120 min after the meal. The EGCG treatment attenuated postprandial triglycerides (*p* = 0.029) and decreased high-density lipoprotein cholesterol (HDL-c) (*p* = 0.016) at 120 min. No treatment × time interaction was found for total cholesterol, low-density lipoprotein (LDL-c), and glucose or insulin levels. The incremental area under the curve (iAUC) for glucose was decreased by EGCG treatment (*p* < 0.05). No difference was observed in the iAUC for triglycerides and HDL-c. In healthy young women, acute EGCG supplementation attenuated postprandial triglycerides and glucose but negatively impacted HDL-c following a fast-food meal.

## 1. Introduction

In general, fast foods are rich in refined carbohydrates, sodium, and saturated and trans-fatty acids, and they are poor sources of vitamins, minerals, and dietary fibers [1]. The frequent consumption of fast food has been associated with overweight and obesity, cardiovascular disease, type 2 diabetes, and other metabolic disorders [2]. The acute consumption of a high-fat, energy-dense, fast-food meal promotes postprandial impairments of the lipid profile and induces oxidative stress in subjects with metabolic syndrome [3]. These acute metabolic damages are not only found in individuals with chronic diseases. In a crossover study, different types of fast-food meals negatively impacted postprandial lipids, insulin, and flow-mediated endothelium-dependent dilatation in healthy volunteers [4].

Plant polyphenols have antioxidant and anti-inflammatory properties and may be responsible for numerous beneficial effects on human health [5]. Epigallocatechin-3-gallate (EGCG) is the main catechin present in green tea, and it is among the best-studied polyphenols [6]. It has been used in the prevention and treatment of non-communicable chronic diseases [7,8]. Treatment with EGCG for 12 weeks decreased bodyweight, total cholesterol, and low-density lipoprotein (LDL-c) in women with central obesity [8], while acute EGCG supplementation was able to delay gastric emptying and increase adiponectin levels in healthy women [9]. In addition, EGCG was observed to decrease lipid absorption from the gastrointestinal tract, which was associated with improvements in insulin resistance and liver triglyceride concentrations [10]. It is important to mention that a cup of green tea (100 mL), one of the main sources for EGCG, contains approximately 144–150 mg of EGCG [11]; nevertheless, EGCG supplements containing different amounts of this catechin are commercialized and easily found for purchase. 

We hypothesized that the negative impact on the lipid profile promoted by fast-food meals, rich in saturated fatty acids, could be improved by the acute administration of EGCG. This study sought to evaluate the effects of acute EGCG supplementation on the postprandial lipid profile, glucose, and insulin levels following a fast-food meal among young healthy women. Even though chronic administration of EGCG can impact lipid profile and other metabolic markers [12], we were not aware of studies evaluating the EGCG acute impact on lipid profile after a high-fat fast-food meal in young and healthy individuals. Therefore, we believe this study may help in increasing knowledge regarding EGCG’s possible effects on the human lipid profile.

## 2. Materials and Methods 

### 2.1. Study Design and Participants

This was an acute, randomized, double-blind, crossover, placebo-controlled trial with healthy young women, aged 18 to 25 years old. The recruitment of the participants was carried out through social media and fliers placed on notice boards at the university campus. Exclusion criteria included smoking, diagnosis of underweight or overweight as assessed by body mass index (BMI), hypertension, diabetes mellitus, dyslipidemia, heart disease, peripheral vascular disease, a history of liver or kidney disease, the use of anti-inflammatory medications and corticosteroids, dietary restrictions related to the foods used in the study (french fries, bacon, and parmesan cheese), and pregnancy and/or breastfeeding. This study was conducted according to the Declaration of Helsinki and was approved by the Ethics Committee of the Federal University of Goiás (# 2.011.261). Written informed consent was obtained from all participants. This study was registered at http://www.ensaiosclinicos.gov.br/ as RBR-2b8p4n. All participants underwent a baseline medical history screening to determine that they were healthy. 

### 2.2. Experimental Protocol

**Figure 1:**
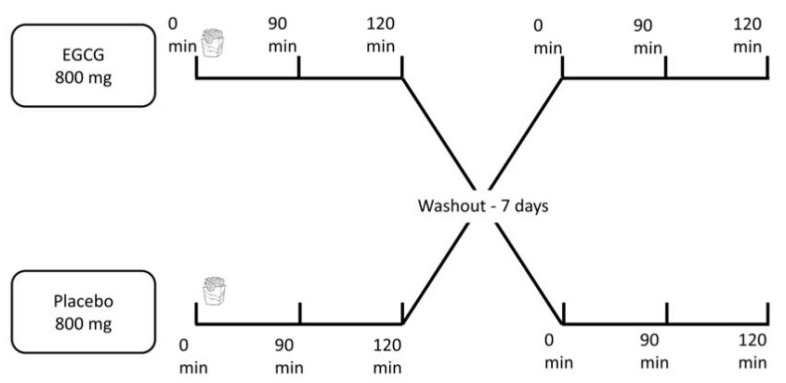
Study design.

A fast-food meal was provided to the participants after a 12 h overnight fast. All participants were instructed to abstain from fresh fruit and vegetables, alcohol, herbal teas, fruit juices, and physical exercise for at least 24 h before the trials, but otherwise were to maintain their regular diet before the two visits, with one week of washout between the sessions [6,13]. Participants were reminded 36 h before each visit about the instructions and were questioned about compliance upon arrival at the laboratory. Right before the high-fat fast-food meal, participants ingested 800 mg, split into two capsules of 400 mg each, of either EGCG or corn starch (placebo). The capsules for the placebo and the active product were similar in appearance, smell, and taste. As presented in a review of the literature performed by our team, studies have used a very wide range regarding amounts of EGCG used in clinical trials [12]. The decision to use a high EGCG dose was based on the positive effects previously found by our research group [9] and the fact that the chronic use of this amount has not promoted harmful side effects [14]. Blood samples were collected at baseline and then 90 and 120 min after the end of the fast-food meal (Figure 1). The times at 90 and 120 min for blood collection were chosen taking into consideration the fact that green tea polyphenols reach peak serum concentrations in the range of 1.3 to 1.6 h [6].

The fast-food meal consisted of 80 g of McDonald’s^®^ french fries, 130 g of bacon cooked for 5 min without the addition of oil, and 60 g of grated Parmesan cheese. The meal contained 20.57 g of carbohydrate, 47.41 g of protein, and 83.90 g of fat, of which 37.17 g was saturated fat, adding to a total of 1027 kcal. This meal was chosen because of its popularity in pubs and fast-food restaurants in Brazil and other countries. Habitual dietary intake was gathered from all involved in the study using a 24 h recall including three non-consecutive days: two weekdays and one weekend day.

### 2.3. Anthropometric and Body Composition Assessments

Body weight, height, and waist circumference were measured according to the procedures described by Lohman (1988) [15], using a digital anthropometric scale and a stadiometer (Filizola^®^, São Paulo, Brazil). All of the measurements were done in duplicate, and the mean between the two findings was used. These values were used to calculate the body mass index (BMI) (kg/m^2^). The estimations of their lean mass values and body fat percentage were assessed by dual-energy X-ray absorptiometry (DXA, Lunar DPX NT, GE Healthcare^®^) with the enCORE 2011 software (version 13.60).

### 2.4. Analyses of Samples

Blood samples were collected from the antecubital vein and immediately centrifuged at 4000 rpm for 10 min at 4 °C in a refrigerated centrifuge (Hitachi CF16RN, Hitachinaka, Ibaraki, Japan) to obtain the serum. The serum was immediately frozen and stored at −80 °C until analysis. Glucose concentrations were determined by the enzymatic colorimetric method. Total cholesterol (TC), high-density lipoprotein cholesterol (HDL-c), triglycerides (TG), and insulin were determined by immunoturbidimetry methods (Architect Plus^®^, Naperville, IL, USA). The concentration of low-density lipoprotein (LDL-c) was calculated using Friedewald’s equation, and VLDL-c dividing TG by five [16]. 

### 2.5. Statistical Analyses

Sample size calculation was performed using G*Power software version 3.1.9.2, taking into consideration the effect on TG [17]. With an effect size of 1.81, a level of significance of 5%, and a statistical power of 95%, the minimum required sample was seven individuals in each treatment. Software R and RStudio were used for the statistical analyses. Values are presented as mean and standard error. The analyses were conducted considering the variations at the timepoints of 90 and 120 min after supplementation and the ingestion of the fast-food meal concerning the baseline measures of the study. The analysis of normality of the residues was performed by the Lilliefors test for the study variables. The carryover effect was analyzed as explained by Rosner (2011) [18], with no significant values. Effect sizes (ES) were calculated using Cohen’s formula and classified as small (d = 0.2), medium (d = 0.5), or large (d = 0.8). Mean tests were performed by analysis of variance (factorial ANOVA) and the differentiation test of Tukey. Postprandial responses between treatments were also compared with the incremental area under the curve (iAUC) using the trapezoidal method [19]. The significance level of 5% was adopted for all tests.

## 3. Results

**Figure 2:**
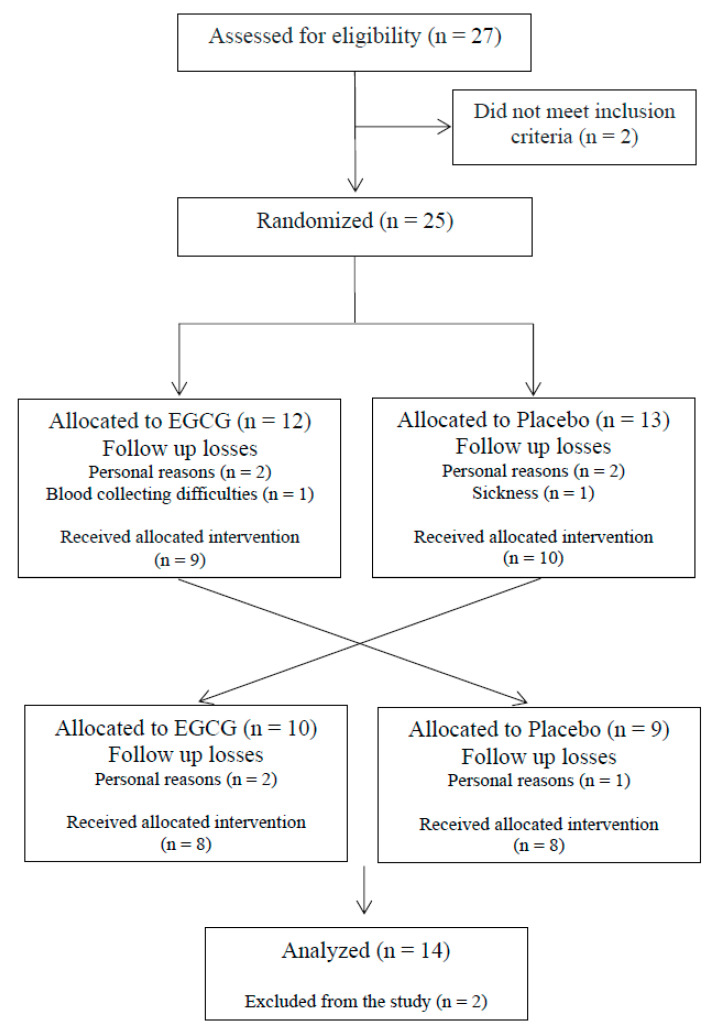
Flow diagram.

Twenty-seven participants were assessed for eligibility; however, only 25 were randomized into the treatments due to the use of anti-inflammatory drugs or BMI. During the study, nine participants dropped out due to personal reasons (*n =* 7), blood collecting difficulties (*n =* 1) and sickness (*n =* 1), and two participants were excluded from the study due to hypertriglyceridemia (Figure 2). The mean age and BMI were 21 ± 1 years of age and 21.4 ± 0.41 kg/m^2^, respectively. The baseline characteristics of the participants are described in Table 1. 

A significant treatment × time interaction was found for TG, VLDL-c, and HDL-c (*p* < 0.05, Table 2). The EGCG group attenuated the increase in TG compared to the placebo at 120 min (+36.1 ± 6.25 vs. +45.9 ± 6.46 mg/dL, respectively, *p* = 0.029, ES = 0.33). VLDL-c concentrations followed the same pattern. Participants from the EGCG group also had greater reductions of HDL-c concentrations (EGCG: −6.5 ± 0.72 vs. placebo −.50 ± 0.44 mg/dL, *p* = 0.016, ES = 0.80) compared to the placebo group at 120 min (Table 2). There was no significant difference in LDL-c. There was a marginal significance in attenuation of postprandial glucose at 90 min (*p* = 0.061, ES = 0.71) and 120 min (*p* = 0.094, ES = 0.24) in the EGCG group compared to the control. The iAUC for glucose (*p* = 0.047) was lower in the EGCG group compared to the placebo (Table 2). No unintended effects were observed throughout the experiment that were related to the ingestion of the fast-food meal or the EGCG supplementation.

## 4. Discussion

To our knowledge, this is the first trial that evaluated the acute impact of EGCG on lipid profile after a high-fat fast-food meal in young and healthy individuals. In the present study, acute administration of EGCG attenuated postprandial TG; however, it also promoted a larger decrease in HDL-c after the high-fat fast-food meal. Considering that this type of food is frequently consumed by young people [20] and that exaggerated postprandial hypertriglyceridemia is a risk factor for cardiovascular disease [21,22], the results of this study might have important applicability in clinical practice.

Corroborating with our results, a similar finding was observed when male adults with borderline and mild hypertriglyceridemia (range 122.12–220.35 mg/dL) submitted to acute ingestion of different amounts of catechin extract from green tea (moderate dose = 224 mg and high dose = 674 mg) [23]. After a light meal, the postprandial TG curves decreased by 15.1% and 28.7%, respectively, compared to the control group [23]. Here we did not find an effect on the iAUC for triglycerides, which may be related to the fact that our study was dealing with younger and healthier individuals. This suggests that EGCG administration can be especially important for populations at high risk of coronary heart disease, considering that postprandial lipidemia is an independent risk factor for cardiovascular diseases [24] and might be predictive of an elevated risk of myocardial infarction [25].

Chronic interventions using EGCG have also shown postprandial TG decreases in mice fed a high-fat diet [26] and in obese patients [27,28]. In a randomized, double-blind, placebo-controlled study, the consumption of a green tea extract containing 208 mg of EGCG associated with minerals decreased BMI, waist circumference, TC, and LDL-c after three months in obese patients [27]. These results for the lipid profile corroborate findings obtained in our previous study with overweight women taking 1 g/day of green tea extract (560 mg polyphenols, ~224 mg of EGCG) for three months [28]. In the present study, TC and LDL-c were not acutely affected by the one-time EGCG intervention. It has been reported that a postprandial increase in TG was associated with a decrease in LDL-c among men newly diagnosed with metabolic syndrome [29], which was also observed in the current study. Thus, the effects of EGCG on LDL-c seem to occur in a chronic rather than an acute manner. 

Huang et al., 2018 [30] suggested that the impact of EGCG on lipid absorption is due to a decrease in bile acid reabsorption, which is necessary for the digestion and metabolism of lipids [31]. A decrease in postprandial HDL-c may also occur when TG is reduced, and its plasma concentration should drop rapidly, considering that HDL-c concentration is dependent on the metabolism of TG-rich lipoproteins [32]. If less fat is absorbed after EGCG administration, this polyphenol may be an important tool to decrease the postprandial deleterious effects of high-fat meals. 

In an experimental study conducted with male C57BL/6J mice, the benefits of EGCG for alleviating insulin resistance and liver TG concentration have been attributed to decreased lipid absorption and reduced inflammatory cytokine concentrations [10]. In our study, the iAUC for glucose decreased in the EGCG group, which was not observed for postprandial insulin concentrations. This might be due to the acute design of the study or because the participants were healthy. It has been reported that individuals with poor fasting blood glucose control show a greater decrease in glucose and insulin concentrations when submitted to dietary interventions [33]. Thus, we might speculate that the intervention attenuates negative effects of a high-fat meal, which should be confirmed in future clinical trials. In a counter-balanced crossover design with 12 healthy men, the administration of green tea extract (containing ~366 mg of EGCG) 24 h before the oral glucose tolerance test increased insulin sensitivity by 13% [34]. In addition, the consumption of 1.5 g of green tea diluted in 150 mL of hot water decreased glucose levels at 30 and 120 min after the oral glucose tolerance test in healthy humans compared to the control group [35]. The authors did not measure the EGCG amount; however, based on previous studies [36,37], it is expected to be around 663 mg of EGCG. Hence, additional studies should be conducted with different populations and EGCG doses. The results obtained in the current study may contribute to the investigation of potential acute benefits for the use of EGCG.

Concerning the limitations of this study, the lack of biomarkers of oxidative stress, measurement of lipid absorption, and postprandial lipemia up to 4 h after the meal should be mentioned. Nevertheless, Kriketos et al., 2003 [17] and Unno et al., 2005 [23] showed the largest increase in postprandial TG occurring 2 h after a high-fat meal, and no increase was found after 3 h. It is expected that the impact of EGCG may continue in the following hours since this catechin would remain at fairly high levels in the bloodstream for 4 h after consumption of 800 mg of EGCG [14]. Besides, we only tested one type of meal, and the effects of EGCG after low-fat or standard meals may differ considerably. 

## 5. Conclusions

We found in healthy young women that EGCG attenuated postprandial TG and glucose concentrations but negatively impacted HDL-c following a high-fat fast-food meal. Our results reinforce that even when administered acutely, EGCG may have an impact on the lipid profile. However, further studies are required to elucidate the effects of EGCG after fast-food meals on cardiovascular risk markers.

## Figures and Tables

**Table 1:** Baseline characteristics of participants.

Variables	Mean ± SEM *n =* 14
**Anthropometry and Body Composition**	
Height (m)	1.6 ± 0.02
Weight (kg)	54.9 ± 1.21
BMI (kg/m^2^)	21.4 ± 0.41
Waist circumference (cm)	67.5 ± 0.91
Lean mass (kg)	34.4 ± 0.99
Fat mass (kg)	16.8 ± 0.87
Fat mass (%)	32.8 ± 1.42
**Food Intake**	
Calories (kcal)	1619 ± 138
Carbohydrate (g)	200 ± 22.8
Carbohydrate (%)	49.1 ± 2.33
Protein (g)	79.4 ± 7.97
Protein (%)	22.9 ± 2.83
Fat (g)	49.3 ± 4.55
Fat (%)	27.98 ± 2.02
Cholesterol (mg)	460 ± 57.7
Total fiber (g)	16.1 ± 2.52
**Biochemical analyzes**	
Total cholesterol (mg/dL)	152 ± 7.12
HDL-c (mg/dL)	59 ± 3.28
LDL-c (mg/dL)	76.8 ± 6.4
Triglycerides (mg/dL)	83.3 ± 7.17
VLDL-c (mg/dL)	16.6 ± 1.19
Blood glucose (mg/dL)	89.5 ± 0.89
Insulin (µU/mL)	7.89 ± 0.81
HOMA-IR	1.74 ± 0.18

HOMA-IR: homeostatic model assessment for insulin resistance.

**Table 2:** The response of the epigallocatechin-3-gallate supplementation after a high-fat fast-food meal.

	Placebo	EGCG
**Total cholesterol at baseline (mg/dL)**	152 ± 21.2	151 ± 5.88
Δ 90	−2.93 ± 1.34	−4.07 ± 1.65
Δ 120	−7.86 ± 1.73	−11.14 ± 1.69
iAUC	−293 ± 99.2	−411 ± 115
**HDL-c at baseline (mg/dL)**	59.3 ± 3.17	60 ± 3.44
Δ 90	−2.64 ± 0.47	−3.64 ± 0.68
Δ 120	−4.50 ± 0.44	−6.5 ± 0.72 *
iAUC	−226 ± 32.0	−316 ± 48.3 ^†^
**LDL-c at baseline (mg/dL)**	76.2 ± 6.87	73.0 ± 6.14
Δ 90	−9.36 ± 1.37	−8.77 ± 1.13
Δ 120	−12.5 ± 1.61	−11.9 ± 1.40
iAUC	−749 ± 103	−704 ± 78.1
**Triglycerides at baseline (mg/dL)**	84.2 ± 7.57	89.9 ± 9.21
Δ 90	45.4 ± 5.92	41.7 ± 7.49
Δ 120	45.9 ± 6.46	36.1 ± 6.25 *
iAUC	3409 ± 446	3045 ± 518
**VLDL-c at baseline (mg/dL)**	16.8 ± 1.51	18.0 ± 1.84
Δ 90	9.07 ± 1.18	8.34 ± 1.50
Δ 120	9.17 ± 1.42	7.23 ± 1.16 *
iAUC	681 ± 89.4	609 ± 103
**Blood glucose at baseline (mg/dL)**	88.4 ± 1.19	90.8 ± 1.27
Δ 90	−0.86 ± 1.31	−5.78 ± 1.89 ^†^
Δ 120	2.57 ± 1.59	−0.07 ± 1.58 ^†^
iAUC	−12.86 ± 94.6	−348 ± 122 *
**Insulin at baseline (µU/mL)**	6.59 ± 0.77	9.59 ± 0.99 *
Δ 90	15.4 ± 2.83	10.5 ± 2.63
Δ 120	17.9 ± 3.45	13.6 ± 2.85
iAUC	1191 ± 214	835 ± 192

Values expressed as means and standard error of means ± SEM. EGCG: epigallocatechin-3-gallate. iAUC: the incremental area under the curve. ^†^ *p* = 0.05 to < 0.10 (marginal significance) and * *p* < 0.05 between treatments analyzed by factorial ANOVA test.
